# β-Defensin-1 Regulates Influenza Virus Infection in Human Bronchial Epithelial Cells through the STAT3 Signaling Pathway

**DOI:** 10.3390/pathogens12010123

**Published:** 2023-01-11

**Authors:** Sreekumar Othumpangat, John D. Noti

**Affiliations:** Allergy and Clinical Immunology Branch, Health Effects Laboratory Division, National Institute for Occupational Safety and Health, Centers for Disease Control and Prevention, Morgantown, WV 26505, USA

**Keywords:** *DEFB1*, bronchial epithelial cells, STAT pathway, influenza virus, miRNA

## Abstract

Understanding the host response to influenza A virus (IAV) infection is vital for developing intervention strategies. The primary barriers for invading respiratory pathogens are the respiratory tract epithelial cells and antimicrobial proteins generated by these cells. The antimicrobial peptide, β-defensin-1, has antiviral activity against both enveloped and non-enveloped viruses. Significant downregulation of β-defensin1 gene (*DEFB1*) expression was observed when human bronchial epithelial cells (HBEpCs) were exposed to IAV. HBEpCs overexpressing *DEFB1* caused a significant reduction in IAV, that was confirmed by IAV matrix gene analysis, plaque assay, and confocal microscopy. *DEFB1* expression after transfection with two micro RNAs (miRNAs), hsa-miR-186-5p and hsa-miR-340-5p, provided evidence that *DEFB1* expression could be modulated by these miRNAs and hsa-miR-186-5p had a higher binding efficiency with *DEFB1*. Overexpression of *DEFB1* in IAV-infected HBEpCs led to increased NF-κB expression. In a PCR array analysis of 84 transcription factors, either overexpressing *DEFB1* or siRNA silencing of *DEFB1* expression significantly modulated the expression of signal transducer and activator of transcription 3 (STAT3). In addition, Ingenuity Pathway Analysis (IPA) integrated with PCR array data showed that the JAK1/STAT3 pathway was significantly altered in cells overexpressing *DEFB1*, suggesting this to be one of the pathways by which defensin regulates IAV replication in HBEpCs. In conclusion, the reduction in IAV copy number in *DEFB1* overexpressing cells suggests that β-defensin-1 plays a key role in regulating IAV survival through STAT3 and is a potential target for antiviral drug development.

## 1. Introduction

The primary barriers for invading pathogens are the airway epithelial cells of the respiratory tract and the antimicrobial peptides produced by these cells. Of these antimicrobials, defensins have a significant role in protecting the cells from pathogens. Defensins are a family of 3–5 kDa cationic peptides rich in cysteine disulfide bonds [1] with a dominant β-sheet structure and three intramolecular disulfide bridges [2] and are part of the innate and adaptive immune system. Defensins are divided into three subfamilies, α, β, and ð defensins, based on their cysteine pairing and molecular structure. Human β-defensin-1 (hBD1) has antiviral activity against both enveloped and non-enveloped viruses [3]. HBD1 is encoded by the gene *DEFB1* and produced by different epithelial and bone-marrow-derived cells that have antimicrobial activity against several different types of pathogens, including bacteria, viruses, fungi, and protozoa [1,3,4]. There are over 28 β-defensin genes identified in humans [5]. HBD1 is primarily expressed by epithelial cells at mucosal surfaces, such as those in the gut, skin, airway, mouth, kidney, nose, eyes, mammary glands, and female and male genital tracts [3,6,7]. HBD1 is constitutively produced by epithelial cells and is reported as a potential mediator in mucosal immunity of the lower respiratory tract [8,9]. HBD1 is also expressed in the respiratory epithelium of the lungs and protects the airways against respiratory pathogens [10]. Infections of the respiratory tract by viruses are one of the major causes of morbidity and mortality in humans, and hBD has shown significant antiviral activity against influenza A virus (IAV), respiratory syncytial virus (RSV), and rhinovirus (RV) [11,12]. Antiviral inactivation of these peptides involves multiple mechanisms, such as direct binding of the virus to the peptide and indirect methods of inactivation through the modulation of viral replication, or signaling pathways, and recruitment of immune cells [12].

IAV, which belongs to the Orthomyxoviridae family, is a human respiratory pathogen that causes both seasonal and periodic pandemics [13]. IAV pandemics can be responsible for substantial morbidity and mortality, particularly in high-risk groups. Antiviral response factors present in host tissues possess the ability to reduce viral replication. An increase in inflammatory cytokines is well documented during influenza infections [14]. An RNA interference study showed the dependency of the influenza virus on host factors for replication and survival, and approximately 295 cellular factors were identified as potentially involved in the initial phase of viral multiplication in host cells [15]. Furthermore, several host cell genes are required for IAV survival and replication [16]. IAV induces the production of hBD2 in epithelial cells of the respiratory tract both in vivo and in vitro [7]. In another in vitro study, Madin–Darby canine kidney (MDCK) cells infected with IAV were protected by blocking entry of the virus with recombinant murine β-defensin-2 [17].

MicroRNAs (miRNAs) are small non-coding RNAs that have a significant role in regulating the pathology of infectious viral diseases. MiRNAs are regulators of gene expression, and their increase or decrease in expression alters the down- or upregulation of protein synthesis. Target gene expression is generally repressed through translational inhibition and RNA degradation. Our earlier studies [18,19] have indicated that miRNA profile was significantly changed on exposure to influenza virus in human airway epithelial cells. The miRNA-mediated regulation of hBD1 is still obscure and, hence, studies were conducted to identify the miRNAs regulating the hBD1 synthesis.

As IAV undergoes antigenic shift and drift, vaccines become less effective, and antiviral peptides, such as hBD1, produced by the host are an important line of defense against infection. The function of hBD1 is not well explored in HBEpCs during IAV infection. Here, we provide evidence that microRNAs miR-186-5p and miR-340-5p regulate expression of hBD1. We show that upregulation of hBD1 leads to upregulation of the transcription factor STAT3 and decreased replication of IAV.

## 2. Materials and Methods

### 2.1. Propagation of Cells

HBEpCs were obtained from PromoCell (Heidelberg, Germany) and sub-cultured in airway epithelial cell growth media (cat # C-21060) and growth factors following supplier recommendations. Madin–Darby canine kidney (MDCK) cells were cultivated in Eagle’s Minimum Essential Medium (MEM, ATCC), adding 10% fetal bovine serum, penicillin, and streptomycin sulfate (100 µg/mL each). MDCK cells were used for the cultivation of influenza viruses A(H1N1) (A/WSN/33), A(H9N1) A(IWF10), and A(H9N1) (IP10). A(H1N1) (A/WSN/33) was provided by Prof. Robert A. Lamb (Northwestern University, Chicago, IL, USA), and the A(H9N1) viruses were from Daniel Perez (University of Georgia, Athens, GA, USA). Cultivation and maintenance of the viruses were carried out as described [19,20,21].

### 2.2. Viral Infections

All infections of HBEpCs were performed in 6-well plates at different multiplicity of infection (MOI) as specified for each experiment. Control cells were mock-infected and described as “mock”. Six-well plates were seeded with 1 × 10^5^ cells per well and grown to 80% confluence. The cells were prerinsed with phosphate-buffered saline (PBS) prior to infection experiments, and Modified Hank’s buffer salt solution (MHBSS) was used for diluting the virus to obtain specific MOI. After 45 min of incubation, excess virus was washed off using PBS and then DMEM/F12 media (ATCC) was added with (A(H9N1) strains) or (A(H1N1) (A/WSN/33) without 1.0 µg/mL of TPCK-trypsin (Sigma-Aldrich, St Louis, MO, USA), and were incubated at 35 ± 1 °C and 5% CO_2_.

### 2.3. hBD1 Overexpression Studies

HBEpCs were transfected with the open reading frame of *DEFB1* cloned in pCMV6-Entry vector (Origene, Rockville, MD, USA) and lipofectamine 2000 (Thermo Fisher Scientific, Carlsbad, CA, USA). Controls were cells transfected with an empty pCMV6 vector. Transfected cells were incubated for 48 h, and then infected with IAV at different MOI. Infected cells were incubated for an additional 24 h. Overexpression of *DEFB1* was confirmed by confocal microscopy using anti-hDB1 antibody as described below in Materials and Methods for Microscopy. Cells were harvested at different time points. RNA was isolated and reverse transcriptase polymerase chain reaction was carried out for the matrix copy number (IAV) and the *DEFB1* transcripts as described earlier [21].

### 2.4. Transfection of miRNAs and siRNAs

HBEpC were transfected with miRNA inhibitor oligonucleotide or a mimic oligonucleotide (Thermo Fisher Scientific, Carlsbad, CA, USA) using the lipid-based lipofectamine 2000 reagent diluted in Opti-MEM-I reduced serum medium (Thermo Fisher Scientific) according to the suppliers’ protocol. Briefly, HBEpCs were grown in 6-well plates overnight and the transfection mixture was directly applied to the cells at a final concentration of 25 nM oligonucleotides. Cells were transfected with the same concentrations of scrambled oligonucleotides (Thermo Fisher Scientific) and used as control. The transfected cells were then infected at various MOIs of IAV. Following the incubation, cells were harvested and used for RNA extraction using RNeasy kit (Qiagen, Germantown, MD, USA).

### 2.5. Defensin β-1 Knock-Down Using siRNAs

The interactions between *DEFB1* and transcription factors during influenza A(H1N1) infection were further investigated by silencing *DEFB1* expression in HBEpCs with *DEFB1*.siRNA (Dharmacon, Chicago, IL, USA). Exponentially growing cells were transiently transfected with either 25 nM of *DEFB1*.siRNA or the scrambled siRNA control using lipofectamine 2000 (Thermo Fisher Scientific). Twenty-four hours following transfection, cells were harvested and used for RNA extraction using RNeasy kit.

### 2.6. Plaque Assay for IAV Detection

For viral plaque assay, confluent MDCK cells were propagated in 6-well plates. Confluent monolayers of MDCK cells were prerinsed with PBS and infected with serially diluted cell culture supernatant (800 μL per well) collected from the HBEpC cells exposed to influenza virus. Infected cells were incubated for 45 min at 35 °C. Cells were then washed with PBS and overlayed with 0.6% agarose (Oxoid Ltd., Hampshire, UK) in DMEM/F12 medium, and plates were kept at 35 °C for another 60 h. Following the incubation, cells were treated with 10% formalin and agarose layer was removed. Cells were stained with 1.0% crystal violet and plaque-forming units (PFU) were counted.

### 2.7. Gene Expression and Transcription Factor PCR Array

Total RNA was isolated from HBEpCs using RNeasy kit (Qiagen) and following the protocol of the supplier. Total RNA (1 µg) was converted to cDNA with the High-Capacity cDNA Reverse Transcription Kit (Thermo Fisher Scientific). TaqMan primers for *DEFB1*, *NF-κB*, *BCL-XL,* and glyceraldehyde phosphate dehydrogenase (*GAPDH*) were purchased from Applied Biosystems (Thermo Fisher Scientific, Foster City, CA, USA). Delta-delta Ct (2^−∆∆Ct^) method was used for calculating the fold change in gene expression as described earlier [21]. Fold change is the normalized expression in each test sample divided by the normalized expression in the control sample. Fold change values less than one (the control sample) indicate a downregulation of expression and the fold downregulated is the negative inverse of the fold change. Values greater than one (the control sample) indicate the fold upregulation of expression.

PCR array analysis for expression and identification of different transcription factors that are differentially regulated in response to IAV infection was performed using TaqMan™ Array, Human Transcription Factors (Non-Hox), Fast 96-well (Catalogue #4418784, Thermo Fisher Scientific). Gene expression results were normalized to the housekeeping gene *HPRT1*, and data were analyzed using the supplier’s online software and the 2^−∆∆Ct^ method.

IAV matrix gene expression was quantified using a standard curve with matrix gene-specific primers (Taqman assay) and reported as influenza matrix gene copy number.

### 2.8. Microscopy

*DEFB1* overexpressing HBEpCs were grown on chamber slides (Chamber slide™, Lab-TekII, Thermo Fisher Scientific, Rochester, NY, USA) to reach 80–90% confluency. Subsequently, cells were infected with A(H1N1) for another 18 h. Cells were washed with PBS and treated with 4% formaldehyde (Polysciences Inc., Warrington, PA, USA). Staining of cells was completed with anti-IAV antibodies (MAB8256, Millipore sigma, Billerica, MA, USA), anti-hemagglutinin, anti-influenza PB1, and anti-hBD1 antibodies (Abcam, Waltham, MA, USA) for 1 h, followed by suitable secondary antibodies (Alexa 488 or −555; Thermo Fisher Scientific). Photomicrographs were produced using a Zeiss LSM510 with a 63X oil immersion lens (Carl Zeiss, Obertochen, AG Germany).

### 2.9. Western Immunoblot Analysis

HBEpC cells were transfected with *DEFB1* plasmid or control plasmid for 48 h, cells were then exposed to A(H1N1) for an additional 24 h and protein extracts (30 µg) from the cells were prepared with 30 µL of radioimmunoprecipitation assay (RIPA) buffer (Thermo Fisher Scientific) and analyzed by 10% SDS-PAGE. Separated proteins were transferred to a nitrocellulose membrane (Bio-Rad, Hercules, CA, USA). Membranes were blocked using Odyssey Blocking Buffer (LI-COR Biosciences, Lincoln, NE, USA) and probed with anti-STAT3, anti-phosphorylated-STAT3, and mouse monoclonal anti-GAPDH (Abcam) antibodies. Appropriate IRDye 680 or 800 secondary antibodies (LI-COR Biosciences) were used. Fluorescence detection was performed on the Odyssey Imaging System (LI-COR Biosciences, Lincoln, NE, USA), and the signal intensities of the individual bands were determined. Protein quantitation was carried out using the Image lite software (LI-COR Biosciences).

### 2.10. Argonaute Immunoprecipitation

Co-immunoprecipitation of Argonaute (Ago) proteins bound to miRNA/mRNA complexes is a powerful tool to validate miRNA targets [22]. Ago Immunoprecipitation (Ago-IP) was completed according to the instructions in the Active Motif kit (Active Motif, Carlsbad, CA, USA). Briefly, cells (HBEpCs) were grown in six-well plates. When cells reached 80% confluency, they were transfected with 25 nM mimics of miR-186-5p and miR-340-5p or negative control (scrambled oligonucleotide of mimic) for 24 h. An isotype control of the antibody alone was run in parallel. Following the manufacturer’s protocol, the Ago-IP precipitated complex was collected, and total RNA was extracted using Trizol reagent (Qiagen). High-Capacity cDNA Reverse Transfection Kit (Thermo Fisher Scientific, Carlsbad, CA, USA) was used for cDNA synthesis. Specific primers for *DEFB1* (Taqman primers, Thermo Fisher Scientific) were used for RT-PCR. The data were analyzed by comparing the cells transfected with mimic miRNA or negative control oligonucleotide, and the fold enrichment of *DEFB1* mRNA was calculated as described by the manufacturer.

### 2.11. Ingenuity Pathway Analysis

Data generated from the transcription factor PCR array were used for the Ingenuity Pathway Analysis (IPA) software (IPA, Qiagen, Redwood City, CA, USA) for core mRNA pathway investigation. The software was utilized for the construction of interacting mRNA pathway networks identified within the *DEFB1* overexpressing group and were compared to a control group.

### 2.12. Statistical Analysis

One-way analysis of variance (ANOVA) was used to analyze MOI studies as well as studies of mimic and inhibitor expression, and post hoc pairwise multiple comparisons between means were performed using the Holm–Sidak method with a *p*-value of <0.05 considered statistically significant using Sigma stat for Windows (Systat Software, Chicago, IL, USA).

## 3. Results

### 3.1. Role of Defensin-β1 in IAV Replication

**Figure 1:**
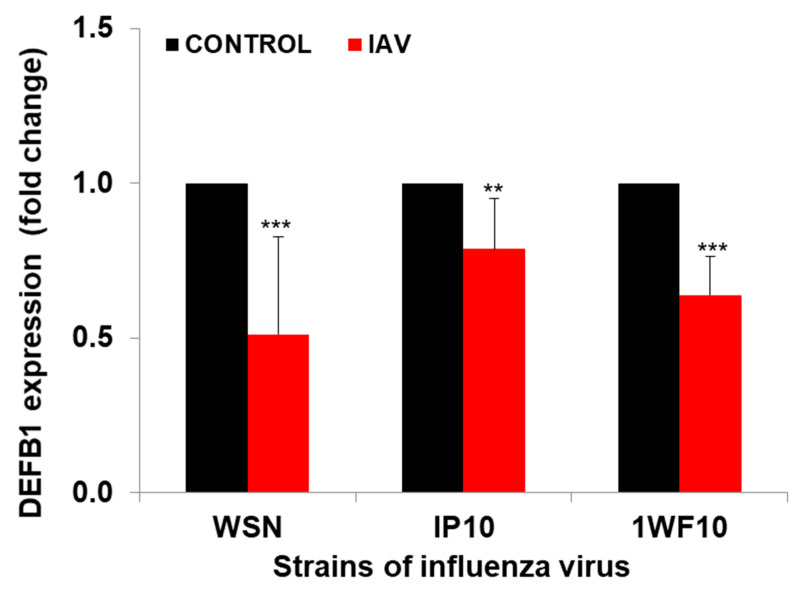
*DEFB1* expression profile. HBEpCs were infected with A(H1N1) and A(H9N1) (1P10 and 1WF10) at MOI of 1.0 for 24 h, and data analyzed by RT-PCR. *n* = 3. *** = *p* < 0.001. ** = *p* < 0.01. Data normalized using the house keeping gene GAPDH.

**Figure 2:**
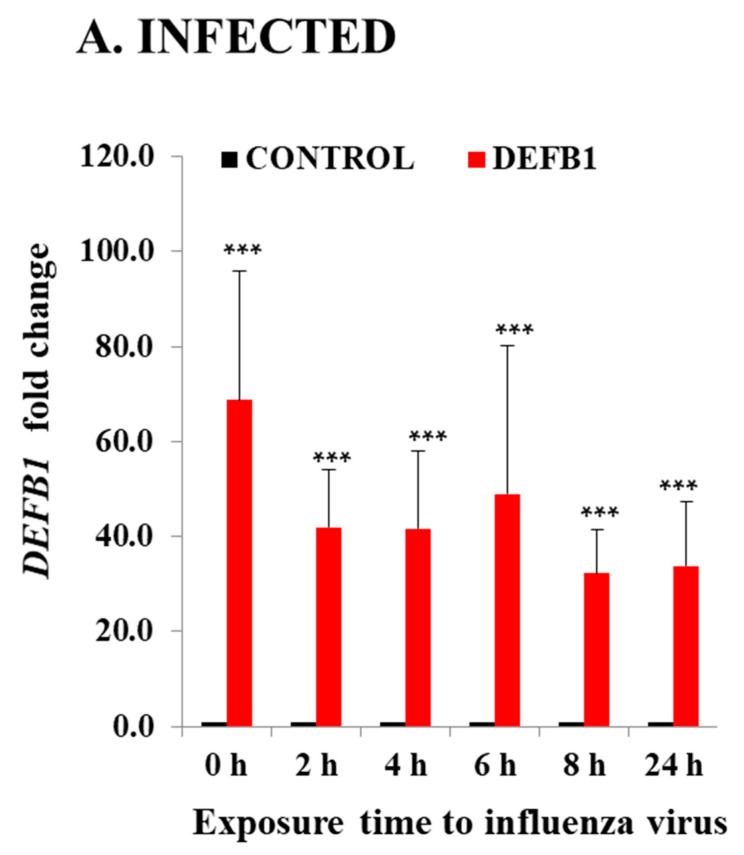

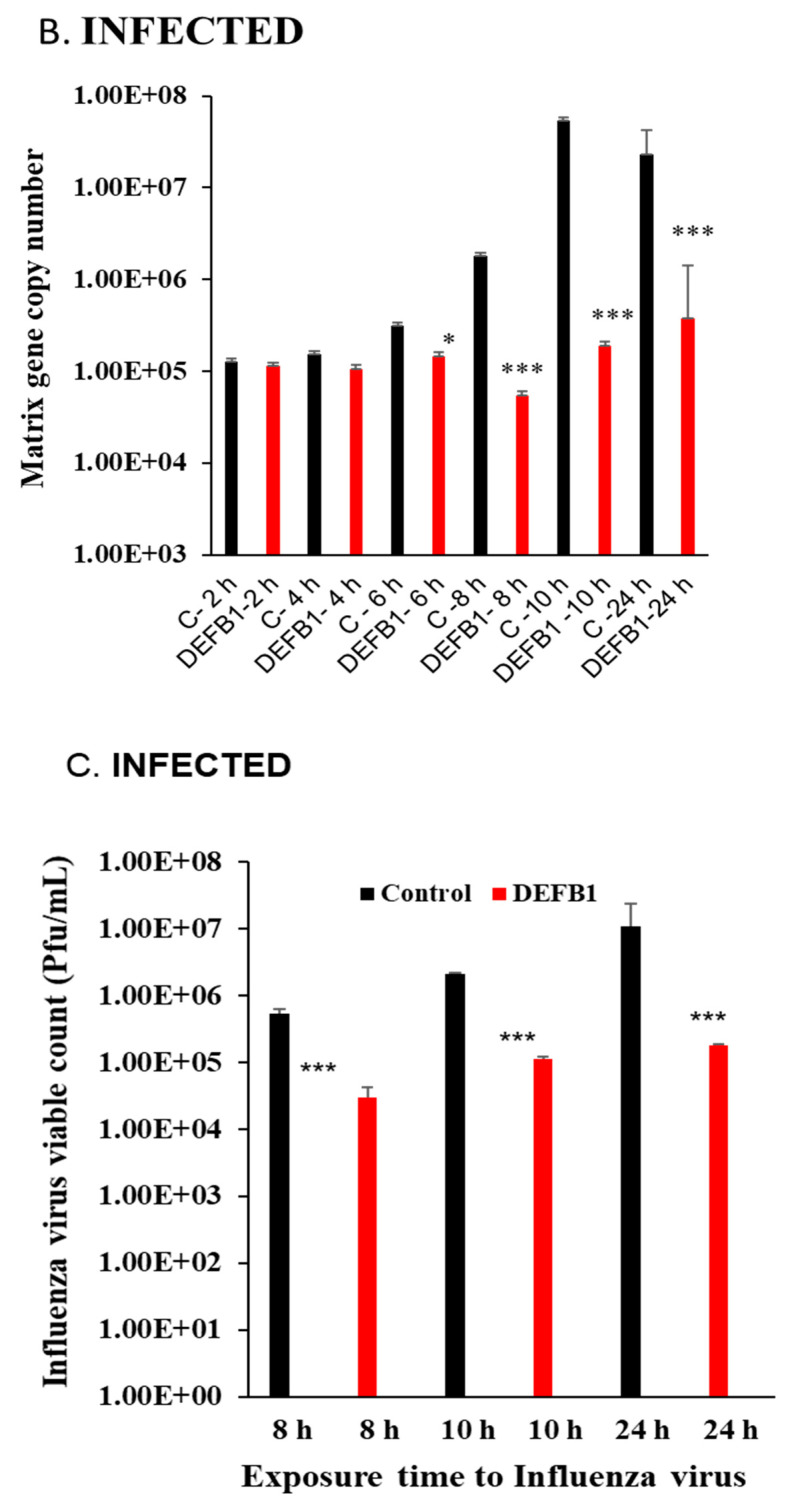
*DEFB1* overexpression protects HBEpCs from A(H1N1) infection. (**A**) HBEpCs were transfected with *DEFB1* plasmid or a control plasmid. Transfected cells were then infected with A(H1N1) at an MOI of 1.0 for 24 h and samples were taken at different time intervals as shown. RNA was extracted and cDNA synthesized and used for RT-PCR analysis. *DEFB1* fold change was calculated using the delta-delta Ct method using GAPDH as housekeeping gene. Data presented as ± SEM, *** = *p* < 0.001, *n* = 3. (**B**) Influenza virus matrix gene determined from the RNA extracted from HBEpCs transfected with *DEFB1* plasmid or control and infected with A(H1N1). Control cells were transfected with the same plasmid that lacks *DEFB1* insert. *n* = 3 experiments., *** = *p* < 0.001, * = *p* < 0.05. (**C**) Viability of influenza virus in cell culture supernatant quantitated as plaque-forming units (pfu/mL). Data shown as mean ± SE (*n* = 3 experiments for plaque assay), *** *p* < 0.001.

Significant downregulation of *DEFB1* expression was observed when HBEpCs were exposed to IAV. The reduction in *DEFB1* was observed with both IAV virus strains A(H1N1) and A(H9N1) indicating that the reduction in *DEFB1* was not strain-dependent (Figure 1). To further understand the role of *DEFB1* during IAV infection, HBEpCs were transfected with either a control plasmid, pCMV6, or pCMV-*DEFB1*, expression of *DEFB1* mRNA was assessed by RT-PCR, and hBD1 protein expression was assessed by confocal microscopy. Both control and *DEFB1* overexpressing cells were then infected with A(H1N1) at an MOI of 1 for 24 h. Samples were drawn at different time intervals (Figure 2). Expression of *DEFB1* in infected cells remained relatively constant over 24 h (Figure 2A). A(H1N1)-infected cells expressing the control plasmid showed increasing A(H1N1) matrix gene copies over 24 h of infection (1 × 10^7.5^ copies), whereas in *DEFB1*-overexpressing cells, matrix gene copies were reduced by 99% (1 × 10^5.5^ copies) (Figure 2B). Our results suggest that *DEFB1* plays a key role in limiting IAV replication in bronchial epithelial cells. In addition, A(H1N1)-infected cells expressing the control plasmid showed increased viable virus in a plaque assay over 24 h of infection, whereas in *DEFB1*-overexpressing cells, the viable virus counts were significantly lower (Figure 2C). Our results suggest that *DEFB1* plays a key role in limiting IAV replication in bronchial epithelial cells.

**Figure 3:**
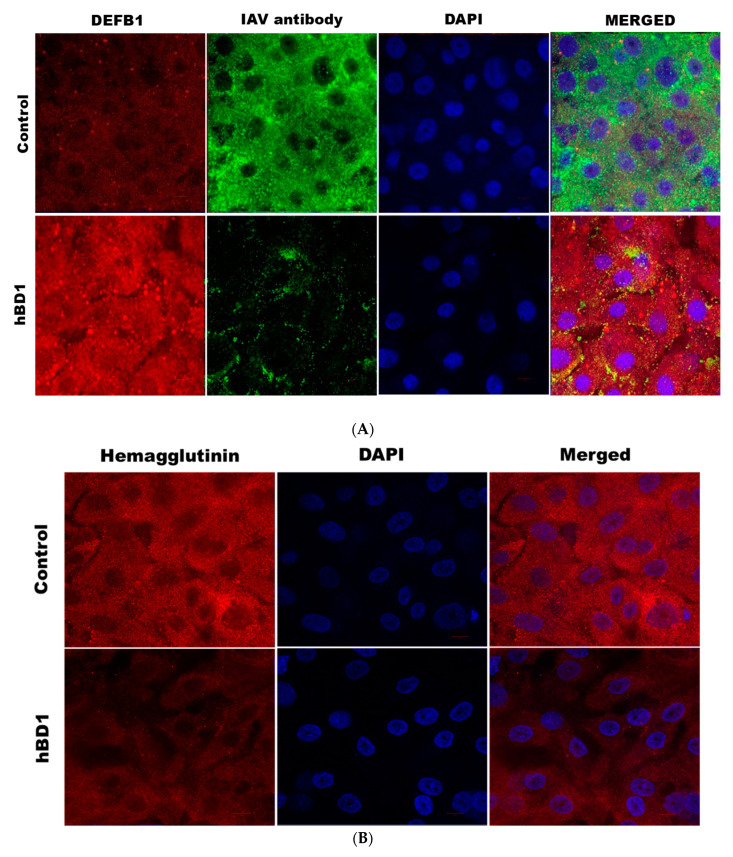

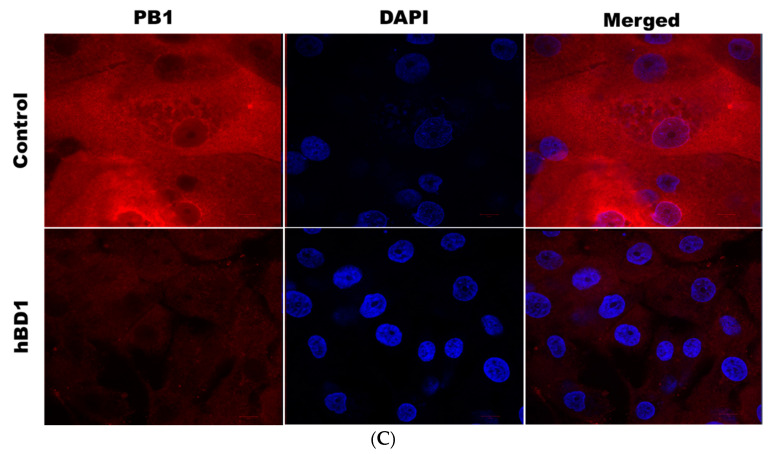
*DEFB1* overexpressing cells show a reduction in different influenza viral proteins. (**A**) HBEpCs overexpressing the *DEFB1* gene or control plasmid were cultured on slides and were infected with A(H1N1) at MOI of 1.0 for 18 h. Cells were stained with anti-hBD1 antibody (red) and IAV antibody MAB8256 (green), and subsequently stained with respective fluorescent secondary antibodies. Nucleus (blue) was stained with DAPI. The microscope settings and contrast adjustments are uniform for a given fluorescence channel across each image. (**B**) Immunofluorescence of hemagglutinin in *DEFB1* overexpressing and control cells. HBEpCs were infected with A(H1N1). DAPI (blue) was used for staining the nucleus of the cells. (**C**) Immunofluorescence of PB1 expression in cells overexpressing *DEFB1* and infected with A(H1N1). Cells were stained DAPI (blue) showing nuclei. All slides were observed using an oil 63X- objective (LSM-510 confocal microscope).

Overexpression of hBD1protein in HBEpCs infected with A(H1N1) was next examined using confocal microscopy (Figure 3A). Cells transfected with the *DEFB1* expression plasmid showed a marked increase in hBD1 expression (shown as increased red fluorescence). HBEpCs transfected with the control plasmid and subsequently infected with A(H1N1) exhibited an intense green fluorescence (IAV antibody) because of active virus replication. In contrast, cells overexpressing hBD1 showed a sharp reduction in IAV infection (Figure 3A, decreased green fluorescence). Decreased IAV infection in cells overexpressing hBD1 was also indicated by a significant reduction in IAV hemagglutinin protein (Figure 3B). An intense overlapping of red fluorescence was visible in control cells infected with A(H1N1) due to the overexpression of influenza virus PB1 protein (Figure 3C) and a decreased detection of PB1 in cells overexpressing hBD1.

**Figure 4:**
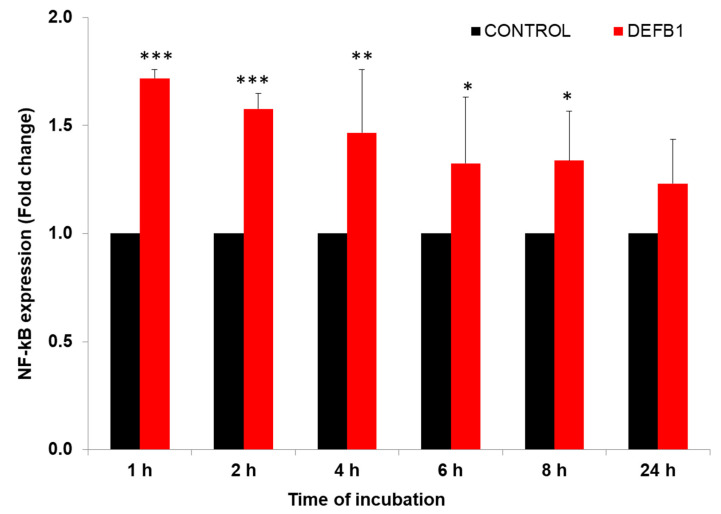
*DEFB1* overexpression modulates the expression of NF-κB. HBEpCs in a 6-well plate were transfected with *DEFB1* or a control plasmid lacking the *DEFB1* gene and infected with A(H1N1) at MOI of 1 for 24 h. Samples were removed at different time intervals for analysis. RT-PCR conducted using extracted RNA from infected cells. NF-κB fold change expression was analyzed using the delta-delta Ct method. NF-κB values are expressed as ± SEM, *** = *p* < 0.001; ** = *p* < 0.01; * = *p* < 0.05. *n* = 3. Control represents the cells transfected with plasmid alone and infected with A(H1N1).

We previously showed that transcription factor NF-κB and its inhibitor NFKBIB regulates IAV replication in HBEpCs [21]. Other studies also have shown that *DEFB1* regulates viral replication through NF-κB [12,23]. HBEpCs were infected with A(H1N1) at an MOI of 1.0 and analyzed NF-κB expression in cells overexpressing *DEFB1* and control plasmid infected cells (Figure 4). Initially (1–2 h post-infection), levels of NF-κB were significantly higher (*p* < 0.001) in *DEFB1* transfected cells. The highest level of NF-κB expression (1.8-fold) was observed in the early stages of A(H1N1) infection and gradually reduced by 8 h (*p* < 0.05) and was not significantly different from that of the control cells by 24 h.

### 3.2. Defensin-β1 Function through STAT3 Pathway

We then used a PCR array consisting of 84 transcription factors that are directly or indirectly involved in viral replication and regulation of the host proteins during IAV infection to search for other transcription factors that may be regulated in response to overexpression of hBD1. Transcription factor expression was first assessed in uninfected HBEpCs that overexpress *DEFB1* and in cells transiently transfected with a combination of three *DEFB1.siRNAs* to downregulate endogenous *DEFB1* expression. HBEpCs that overexpress *DEFB1* showed more than a two-fold upregulation (more than a two-fold change over the plasmid control) of *ATF1, FOXO1, IRF1, RELB, SMAD9*, and *STAT3*, whereas *GTF2B, MEF2C*, and *PPARG* were more than two-fold downregulated (negative fold changes compared with plasmid control) (Table 1).

In contrast, downregulation of endogenous *DEFB1* with siRNAs led to a more than two-fold downregulation of *ATF3, CEBPA, ESR1, HIF1A, MEF2C, NFATC2, PPARG, SP3, STAT1*, and *TFAP2A* expression. Interestingly, not all transcription factors that were upregulated in *DEFB1* overexpressing cells decreased in cells transfected with *DEFB1*.siRNA. For example, *DEFB1*.siRNA transfected cells did not have altered expression of *ATF1, RELB,* and *SMAD9* compared to the control cells.

**Figure 5:**
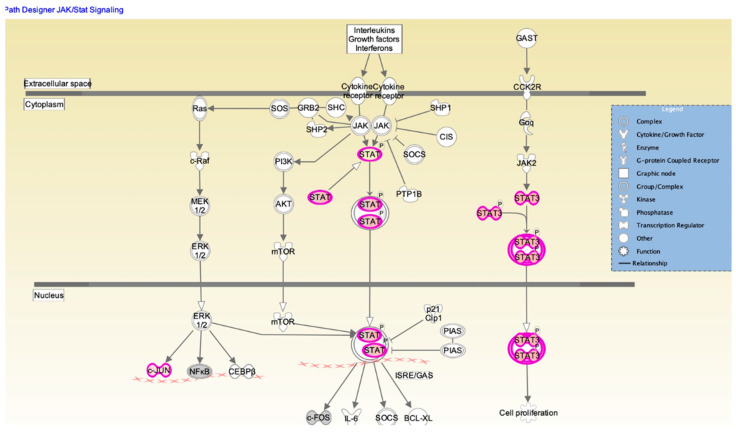
Ingenuity pathway analysis map depicting the STAT/JAK1 signaling pathway. Signaling molecules showing upregulation are indicated by purple and the intensity of purple represents the signal strength. STAT interactions in cytoplasm and nucleus are shown along with other molecules involved in regulating cell proliferation. Biological network analysis was performed using IPA software. Downstream effector molecules, such as *cFOS, IL-6, SOCS*, and *BCl-XL,* are also shown.

**Figure 6:**
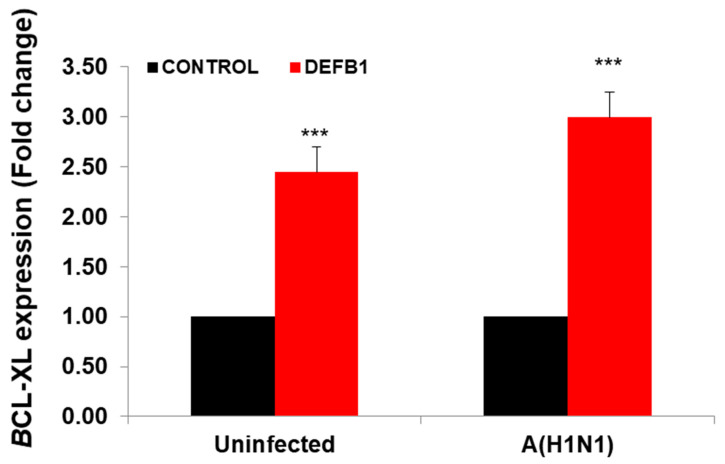
Expression of BCL-XL showing the downstream pathway of STAT/JAK1 in *DEFB1* overexpressing cells. HBEpCs were transfected with *DEFB1* or a plasmid control and infected with A(H1N1) at MOI of 1 and incubated for 24 h. Data are expressed as ± SEM, *** = *p* < 0.001. *n* = 3. Uninfected control is the cells transfected with plasmid alone and not infected with A(H1N1).

**Figure 7:**
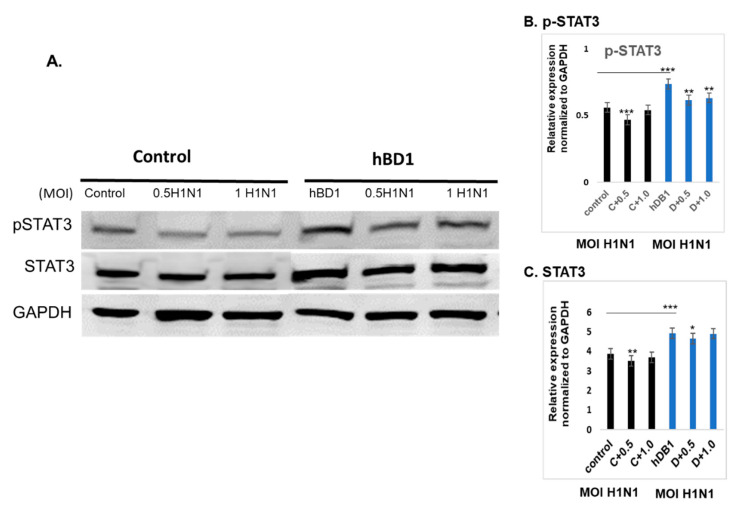
*DEFB1* overexpression modulates STAT3. HBEpCs were transfected with *DEFB1* gene containing plasmid or a control vector plasmid. Transfected cells were incubated for 48 h and the cells were infected with 0.5 and 1 MOI of A(H1N1) for an additional 24 h. (**A**) Western blot of cells overexpressing *DEFB1* showing increased phospho-STAT3 protein in both infected and uninfected cells, whereas the control cells infected with A(H1N1) showed a reduction in STAT3 phosphorylation. Housekeeping protein is GAPDH. (**B**) Quantification of phospho-STAT3, and (**C**) quantification of STAT3. Quantification was performed using the Odyssey lite software. Data are expressed as ± SEM, *** = *p* < 0.001; ** = *p* < 0.01; * = *p* < 0.05. *n* = 3 experiments.

The JAK/STAT signaling pathway is shown in IPA (Figure 5). Changes in cytokine levels during viral infection have been well documented [18] and the IPA showed that interferon and interleukin attachment to cytokine receptors can induce STAT phosphorylation, which in turn modulates expression of downstream genes, such as C-FOS, IL-6, and BCL-XL, along with cell proliferation. We then verified that one of these downstream target genes, BCL-XL, was upregulated in A(H1N1)-infected HBEpCs that overexpress DEFB1 (Figure 6). A significant upregulation of phosphorylated STAT3 in HBEpCs that overexpress DEFB1 was verified by Western blot analysis (Figure 7A). The cells overexpressing DEFB1 (uninfected) showed significant increase in both STAT3 phosphorylation (Figure 7B) and total STAT3 (Figure 7C) The cells overexpressing DEFB1 infected with 0.5 and 1.0 MOI of A(H1N1) also showed significant changes in both STAT3 phosphorylation (Figure 7B) and total STAT3 (Figure 7C).

### 3.3. Role of miRNAs Regulating the Expression of Defensin-β1

**Figure 8:**
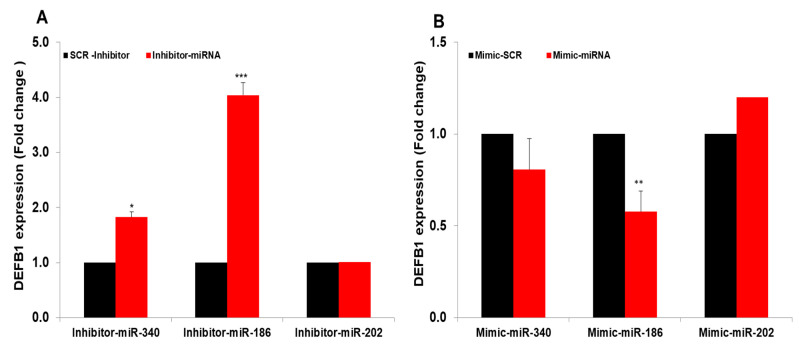
MiRNAs mimics and inhibitors target *DEFB1*. (**A**) HBEpCs were transfected with 25 nM miR-186-5p, miR-340-5p, and miR-202-3p inhibitors or a scrambled oligonucleotide. After 36 h, the *DEFB1* transcript level was determined by RT-PCR. Data presented as ± SEM, *** = *p* < 0.001, and * = *p* < 0.05. (**B**) HBEpCs were grown to confluency (80–90%) in a 6-well plate and then transfected for 48 h with 25 nM miR-186-5p, miR-340-5p, and miR-202-3p mimics or a scrambled oligonucleotide. After 36 h of incubation, RNA was isolated and the *DEFB1* transcript level was determined by RT-PCR. ** = *p* < 0.01.

Our previous studies, along with others, have shown that several miRNAs are differentially regulated during IAV infection of airway epithelial cells [19,21,24]. We therefore assessed whether any of these miRNAs have a role in regulating *DEFB1* expression. Using TargetScan (www.targetscan.org; accessed on 15 August 2021) and miRanda database (version 3.3a 2020, accessed 16, August, 2021), we searched for putative miRNAs that target *DEFB1*. Table 2 shows the top miRNAs that potentially target *DEFB1* mRNA. Of these, we selected three miRNAs, miR-340-5p, miR-186-5p, and miR-202-3p, that were differentially regulated in IAV-infected patient sera in our previous study [18]. HBEpCs were transfected separately with miR-340-5p, miR-186-5p, and miR-202-3p inhibitor oligonucleotides (Figure 8A) or miR-340-5p, miR-186-5p, and miR-202-3p mimic oligonucleotides (Figure 8B). The results showed that miR-186-5p mimic and inhibitor could effectively target *DEFB1* mRNA. MiR-202-3p did not show any inhibition of *DEFB1* with mimic or increase in *DEFB1* expression with inhibitor. MiR-340-5p showed a moderate effect on *DEFB1* expression in cells transfected with inhibitor and mimic oligonucleotides.

**Figure 9:**
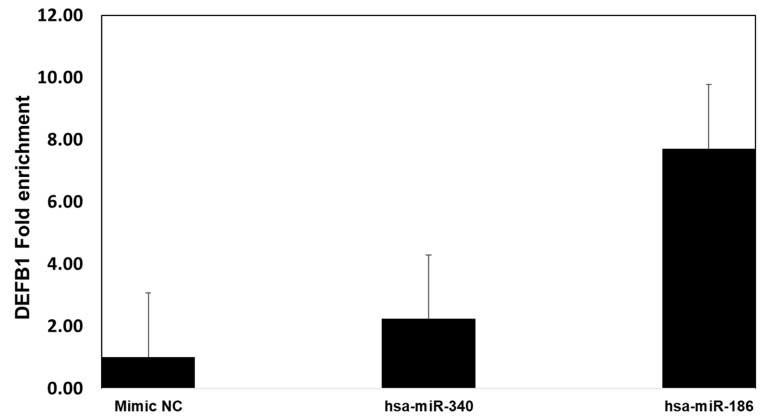
Confirmation of miRNA target of *DEFB1* by Argonaute immunoprecipitation. HBEpCs were transfected with miR-340-5p, miR186-5p mimic, or negative control mimic. Cells were incubated for 24 h. Following the incubation, the cells were lysed, and cell lysate used for immunoprecipitation using the Argonaute 1, 2, 3, or isotype antibody. Fold enrichment of *DEFB1* transcript was determined by extracting RNA and running RT-PCR experiment. The fold enrichment was calculated based on the control cells immunoprecipitated using isotype and ago-antibody. *n* = 2.

To further confirm the specificity in binding of miR-340 and miR-186 to *DEFB1* mRNA, mimics of miR-340-5p, miR-186-5p, or a scrambled oligonucleotide control were used to transfect HBEpCs and then subjected to Argonaute immunoprecipitation (Ago-IP). The results indicate that mimics of miR-186-5p transfected cells showed an 8-fold enrichment of *DEFB1* mRNA compared to the scrambled-mimic-transfected cells (Figure 9). In contrast, miR-340-5p mimic led to only a two-fold enrichment of *DEFB1* mRNA.

## 4. Discussion

Invading pathogens come in direct contact with airway epithelial cells of the respiratory tract where antimicrobial peptides produced by these cells provide an initial defense mechanism. As microbes evolved, these peptides have retained their antimicrobial properties to the microbial target [25]. Several peptides within the β-defensin family have been investigated for their antimicrobial activity against bacteria and fungi [26]. One peptide, hBD1, encoded by *DEFB1*, is widely produced by human secretory glands and epithelial cells [27]. In addition, hBD1 is also expressed in plasmacytoid dendritic cells and monocytes and is induced in response to viral challenge [28]. Andresen et al. [29] studied the modulation of hBD1 expression in Chronic Obstructive Pulmonary Disease (COPD) patients and suggested that hBD1 could be used as a biomarker for COPD. Antimicrobial peptides also play a critical role in mammalian innate immunity. Among the viruses, IAV is a seasonal pathogen that undergoes antigenic drifts that can lower the efficacy of an IAV vaccine, emphasizing the importance of an antimicrobial defense mechanism [3]. Human BD1 plays a significant role in protecting airway epithelial cells from invading viruses and bacteria [11]. Our studies showed that *DEFB1* gene expression is decreased in cells exposed to IAV over a 24 h time period, and the decrease was consistent with both A(H1N1) and A(H9N1) strains. A significant reduction in IAV infection was observed in HBEpCs overexpressing *DEFB1*, suggesting that hBD1, could play a major role in regulating IAV infection in airway epithelium. Our study may help determine specifically how hBD1 regulates IAV replication and can lead to a better understanding of the pathophysiology of IAV infections.

In contrast to our results, expression of sheep BD1 (sBD1) mRNA was increased three days post-infection with IAV [30]. In a mouse model, the first six days of IAV A(H3N2) infection showed increased expression of mouse BD1(mBD1) in the trachea and nasosinuses tissues, whereas mBD1 expression in lung tissues increased in the early course of infection and decreased by days 3 and 6 [31]. The difference in expression of defensins reported in in vivo animal studies and our current studies could be attributed to the viability of the virus, specificity of the virus, and the host cells used. hBD1 showed increased expression in other respiratory viruses, such as rhinovirus infections [23]. In vitro studies in HSV-1-infected airway epithelial cells showed an inverse correlation between *DEFB1* expression and the virus HSV-1 [27]. Our findings supported this conclusion, as overexpression of *DEFB1* significantly reduced IAV copy numbers.

The role of miRNAs in regulating expression of *DEFB1* mRNA has not been reported. We showed that *DEFB1* expression was significantly altered by two microRNAs, miR-186-5p and miR-340-5p. In previous studies, miR-186-5p and miR-340-5p showed differential expression in IAV-infected airway epithelial cells [18,32]. MiRNAs are regulatory RNAs that control the stability of more than half of mRNAs in humans by recruiting the RNA-induced silencing complex through base-pairing interactions with their targets [33]. In most cases, miRNAs modulate gene expression by binding to complementary segments in the 3′UTR regions of the mRNAs. miRNAs could interact with several conserved mRNA targets that can target different signaling pathways [34,35,36]. Our data indicate that mimics and inhibitors of miR-340-5p and miR-186-5p were able to modify the expression of *DEFB1*. Generally, each miRNA can be involved in a very complex network by targeting multiple genes [37]. We demonstrated that the mimics of miR-186-5p were able to reduce *DEFB1* expression, and that this miRNA may have a role in modulating *DEFB1* expression in HBEpCs.

In this study, we also showed that hBD1 can function through the STAT3 pathway. STATs are a family of transcription factors that have vital roles in regulating several biological functions, including immune modulation, angiogenesis, apoptosis, cell differentiation, and induction of inflammatory cytokines [38]. Among the RNA viruses, IAV, human metapneumovirus, and human cytomegalovirus all are able to inhibit phosphorylation of STAT3 [39]. STAT proteins are activated upon phosphorylation and translocate to the nucleus where they activate regulatory genes, cytokines, and growth factors. Our IPA results (Figure 5) support a role for STAT3 in *DEFB1* expression and that *DEFB1* overexpression may function through the STAT3 pathway. STAT3 is activated at Tyr705, and in this study we used an antibody that was specific to detect Tyr705 phosphorylation of STAT3 (Figure 7, Western blot analysis). In our studies, we showed that overexpression of *DEFB1* upregulates STAT3 protein expression and subsequent phosphorylation. Further, our transcription factor array results, which showed increased expression of STAT3 and IRF1 in *DEFB1*-overexpressing cells, suggests that the *DEFB1* may be functional through STAT3 and IRF1, but further studies are needed to confirm this possibility. Combining our previous findings showing that transcription factor *NF-κB* regulates IAV infection (20), we propose that IAV infection suppresses *DEFB1*, and by suppressing *DEFB1* leads to an alteration of *NF-κB* and the subsequent IAV matrix gene, and the downregulation of anti-apoptotic proteins *BCL-XL* and pSTAT3.

## 5. Conclusions

In conclusion, we found that overexpression of *DEFB1,* the gene encoding hBD1, significantly reduced IAV matrix gene copies in human airway epithelial cells. Two miRNAs, miR-186-5p and miR-340-5p, regulate expression of *DEFB1*. The role of STAT3 was further visualized by IPA analysis where the STAT pathway was significantly altered by overexpression of *DEFB1*. The reduction in viable IAV in *DEFB1* overexpressing cells suggests that hBD1 plays an important role in regulating IAV survival through the STAT3 pathway and is a potential target to develop therapies against IAV infections.

## Figures and Tables

**Table 1:** Uninfected HBEpC were transfected with DEFB1 or DEFB1.siRNA showing the expression pattern of transcription factors that were detected. HBEpCs were transfected with DEFB1 and then infected with A(H1N1) showing the expression (fold change) pattern of transcription factors that were detected, along with the house keeping gene HPRT1 (MOI of 1 for 24 h).

Fold Change					
Gene Symbol	Plasmid Control	DEFB1 Control	DEFB1 siRNA	Control+ A(H1N1)	DEFB1+ A(H1N1)
HPRT1	1.00	1.26	0.99	1.00	1.03
HMBS	1.00	1.30	1.40	1.00	0.48
TBP	1.00	1.18	1.03	1.00	0.42
PGK1	1.00	1.07	1.33	1.00	0.71
UBC	1.00	1.71	0.89	1.00	0.41
PPIA	1.00	1.28	1.51	1.00	0.78
ARNT	1.00	1.28	1.24	1.00	0.48
ATF1	1.00	4.26	1.52	1.00	1.76
ATF2	1.00	1.28	0.96	1.00	0.60
ATF3	1.00	1.03	0.42	1.00	3.16
ATF4	1.00	1.32	1.14	1.00	0.33
CEBPA	1.00	1.02	0.42	1.00	0.31
CEBPB	1.00	1.16	0.90	1.00	0.67
CEBPG	1.00	0.60	0.92	1.00	0.14
CREB1	1.00	1.20	0.85	1.00	0.72
CREBBP	1.00	0.97	0.57	1.00	0.42
CTNNB1	1.00	1.48	1.07	1.00	0.84
DR1	1.00	0.92	1.21	1.00	0.33
E2F1	1.00	0.93	1.70	1.00	0.19
E2F6	1.00	1.04	0.74	1.00	0.26
EGR1	1.00	1.13	0.81	1.00	4.95
ELK1	1.00	1.12	0.69	1.00	0.34
ESR1	1.00	1.31	0.36	1.00	0.17
ETS1	1.00	1.66	1.31	1.00	0.29
ETS2	1.00	0.76	1.01	1.00	1.65
FOXA2	1.00	0.65	0.97	1.00	0.28
FOXO1	1.00	3.06	0.97	1.00	0.64
GATA3	1.00	1.19	1.09	1.00	3.01
GTF2B	1.00	0.22	1.59	1.00	0.11
GTF2F1	1.00	1.13	0.79	1.00	0.46
HDAC1	1.00	1.08	1.30	1.00	0.45
HIF1A	1.00	0.95	0.45	1.00	0.09
HSF1	1.00	1.86	1.01	1.00	0.58
ID1	1.00	0.57	1.02	1.00	0.38
IRF1	1.00	2.14	0.92	1.00	2.42
JUN	1.00	0.77	0.93	1.00	1.04
JUNB	1.00	1.48	1.61	1.00	1.44
MAX	1.00	0.97	1.00	1.00	0.64
MEF2A	1.00	1.48	0.77	1.00	0.45
MEF2B	1.00	1.76	1.20	1.00	0.61
MEF2C	1.00	0.07	0.43	1.00	4.19
NFAT5	1.00	1.00	1.26	1.00	0.14
NFATC1	1.00	0.77	0.78	1.00	0.20
NFATC2	1.00	1.12	0.39	1.00	0.39
NFATC3	1.00	1.29	1.13	1.00	0.44
NFATC4	1.00	1.54	1.17	1.00	0.34
NFKB1	1.00	1.88	0.77	1.00	1.10
NFYB	1.00	0.55	0.87	1.00	0.16
NR3C1	1.00	0.76	0.96	1.00	0.49
PPARA	1.00	1.68	0.78	1.00	0.30
PPARG	1.00	0.27	0.28	1.00	0.10
RB1	1.00	1.13	1.01	1.00	0.33
REL	1.00	1.12	1.19	1.00	5.99
RELA	1.00	1.18	1.00	1.00	0.61
RELB	1.00	2.29	1.36	1.00	0.93
SMAD1	1.00	0.98	0.66	1.00	0.31
SMAD4	1.00	0.97	0.92	1.00	0.32
SMAD5	1.00	1.22	1.50	1.00	0.46
SMAD9	1.00	3.36	1.81	1.00	1.93
SP1	1.00	0.88	1.12	1.00	0.38
SP3	1.00	0.68	0.48	1.00	0.26
STAT1	1.00	0.61	0.34	1.00	1.25
STAT2	1.00	0.69	0.74	1.00	2.18
STAT3	1.00	2.03	0.75	1.00	2.16
STAT4	1.00	1.25	1.58	1.00	0.80
STAT5A	1.00	1.01	0.78	1.00	0.65
STAT5B	1.00	1.13	1.01	1.00	0.31
STAT6	1.00	1.68	1.27	1.00	1.07
TBP	1.00	1.37	0.82	1.00	0.42
TCF7L2	1.00	1.43	1.00	1.00	0.40
TFAP2A	1.00	1.56	0.46	1.00	0.64
TGIF1	1.00	1.92	1.77	1.00	1.07
TP53	1.00	0.74	0.79	1.00	0.31

Note: The row highlighted in red displays the relevant genes modulated by *DEFB1*.

**Table 2:** Targeted miRNAs of DEFB1 (data from Target scan).

miRNA	Position at UTR	Seed Match	Context ++Score	Context Percentile	Weighted ++Score	Conserved Branch Length
Hsa-miR-6875-3p	15–21	7mer-1A	−0.16	91	−0.16	0
Hsa -miR-4659b-3p	15–21	7mer-1A	−0.17	91	−0.17	0
Hsa-miR-4659a-3p	15–21	7mer-1A	−0.17	91	−0.17	0
Hsa -miR-4520-5p	24–30	7mer-1A	−0.49	96	−0.49	0
Hsa -miR-5696	35–41	7mer-1A	−0.24	98	−0.24	0
Hsa -miR-664b-3p	35–41	7mer-1A	−0.15	97	−0.15	0.014
Hsa -miR-579-3p	35–41	7mer-1A	−0.15	96	−0.15	0.014
Hsa -miR-6853-3p	36–42	7mer-m8	−0.43	99	−0.43	0
Hsa s-miR-3163	44–50	7mer-1A	−0.07	97	−0.07	0
Hsa -miR-340-5p	45–51	7mer-1A	−0.11	97	−0.11	0.252
Hsa -miR-186-5p	54–60	7mer-m8	−0.26	99	−0.26	0.239
Has-miR-4477a	60–66	7mer-1A	−0.17	97	−0.17	0
Hsa -miR-548e-5p	75–81	7mer-m8	−0.22	98	−0.22	0
Hsa -miR-202-3p	87–93	7mer-m8	−0.45	98	−0.45	0.014
Hsa -miR-3173-3p	90–96	7mer-m8	0.48	99	0.48	0.085
Hsa -miR-6891-5p	90–96	7mer-m8	0.48	99	0.48	0.085
Hsa -miR-186-3p	94–100	7mer-m8	−0.3	99	−0.3	0
Hsa -miR-3136-3p	96–103	8mer	−1.02	99	−1.02	0
Hsa -miR-7155-3p	96–103	8mer	−1.02	99	−1.02	0
Hsa -miR-328-3p	98–104	7mer-1A	−0.44	98	−0.44	0.133

## Data Availability

Not applicable.

## Abbreviations

ARNT	Aryl hydrocarbon receptor nuclear translocator
ATF1	Activating transcription factor 1
ATF2	Activating transcription factor 2
ATF3	Activating transcription factor 3
ATF4	Activating transcription factor 4
CEBPA	CCAAT/enhancer binding protein (C/EBP), alpha
CEBPB	CCAAT/enhancer binding protein (C/EBP), beta
CEBPG	CCAAT/enhancer binding protein (C/EBP), gamma
CREB1	CAMP responsive element binding protein 1
CREBBP	CREB binding protein
CTNNB1	Catenin (cadherin-associated protein), beta 1
DR1	Downregulator of transcription 1, TBP-binding
E2F1	E2F transcription factor 1
E2F6	E2F transcription factor 6
EGR1	Early growth response 1
ELK1	ELK1, member of ETS oncogene family
ESR1	Estrogen receptor 1
ETS1	V-Ets erythroblastosis virus E26 oncogene homolog 1
ETS2	V-Ets erythroblastosis virus E26 oncogene homolog 2
FOS	Fos Proto-Oncogene, AP-1 Transcription Factor Subunit
FOXA2	Forkhead box A2
FOXO1	Forkhead box O1
GATA1	GATA binding protein 1 (globin transcription factor 1)
GATA2	GATA binding protein 2
GATA3	GATA binding protein 3
GTF2B	General transcription factor IIB
GTF2F1	General transcription factor IIF, polypeptide 1, 74 kDa
HAND1	Heart and neural crest derivatives expressed 1
HAND2	Heart and neural crest derivatives expressed 2
HDAC1	Histone deacetylase 1
HIF1A	Hypoxia inducible factor 1, alpha subunit
HNF4A	Hepatocyte nuclear factor 4, alpha
HOXA5	Homeobox A5
HSF1	Heat shock transcription factor 1
ID1	Inhibitor of DNA binding 1
IRF1	Interferon regulatory factor 1
JUN	Jun proto-oncogene
JUNB	Jun B proto-oncogene
MAX	MYC associated factor X
MEF2A	Myocyte enhancer factor 2A
MEF2B	Myocyte enhancer factor 2B
MEF2C	Myocyte enhancer factor 2C
NFAT5	Nuclear factor of activated T-cells 5, tonicity-responsive
NFATC1	Nuclear factor of activated T-cells, calcineurin-dependent 1
NFATC2	Nuclear factor of activated T-cells, calcineurin-dependent 2
NFATC3	Nuclear factor of activated T-cells, calcineurin-dependent 3
NFATC4	Nuclear factor of activated T-cells, calcineurin-dependent 4
NFKB1	Nuclear factor of kappa B 1
NFYB	Nuclear transcription factor Y, beta
NR3C1	Nuclear receptor subfamily 3, group C, member 1
PPARA	Peroxisome proliferator-activated receptor alpha
PPARG	Peroxisome proliferator-activated receptor gamma
RB1	Retinoblastoma 1
REL	REL V-rel reticuloendotheliosis viral oncogene homolog
RELA	V-rel reticuloendotheliosis viral oncogene homolog A
RELB	RELB V-rel reticuloendotheliosis viral oncogene homolog B
SMAD1	SMAD family member 1
SMAD4	SMAD family member 4
SMAD5	SMAD family member 5
SMAD9	SMAD family member 9
SP1	Sp1 transcription factor
SP3	Sp3 transcription factor
STAT1	Signal transducer and activator of transcription 1,
STAT2	Signal transducer and activator of transcription 2,
STAT3	Signal transducer and activator of transcription 3
STAT4	Signal transducer and activator of transcription 4
STAT5A	Signal transducer and activator of transcription 5A
STAT5B	Signal transducer and activator of transcription 5B
STAT6	Signal transducer and activator of transcription 6
TBP	TATA box binding protein
TCF7L2	Transcription Factor 7 Like 2
TFAP2A	Transcription factor AP-2 alpha
TGIF1	TGFB-induced factor homeobox 1
TP53	Tumor protein p53
